# Physiological and Pathological Roles of Aldose Reductase

**DOI:** 10.3390/metabo11100655

**Published:** 2021-09-27

**Authors:** Mahavir Singh, Aniruddh Kapoor, Aruni Bhatnagar

**Affiliations:** 1 Eye and Vision Science Laboratory, Department of Physiology, School of Medicine, University of Louisville, Louisville, KY 40202, USA; 2 Internal Medicine—Critical Care, School of Medicine, Saint Louis University, St. Louis, MO 63141, USA; aniruddhkapoor@gmail.com; 3 Christina Lee Brown Envirome Institute, School of Medicine, University of Louisville, Louisville, KY 40202, USA; aruni.bhatnagar@louisville.edu

**Keywords:** aldose reductase, diabetes, inhibitors, inflammation, diseases, oxidative stress

## Abstract

Aldose reductase (AR) is an aldo-keto reductase that catalyzes the first step in the polyol pathway which converts glucose to sorbitol. Under normal glucose homeostasis the pathway represents a minor route of glucose metabolism that operates in parallel with glycolysis. However, during hyperglycemia the flux of glucose via the polyol pathway increases significantly, leading to excessive formation of sorbitol. The polyol pathway-driven accumulation of osmotically active sorbitol has been implicated in the development of secondary diabetic complications such as retinopathy, nephropathy, and neuropathy. Based on the notion that inhibition of AR could prevent these complications a range of AR inhibitors have been developed and tested; however, their clinical efficacy has been found to be marginal at best. Moreover, recent work has shown that AR participates in the detoxification of aldehydes that are derived from lipid peroxidation and their glutathione conjugates. Although in some contexts this antioxidant function of AR helps protect against tissue injury and dysfunction, the metabolic transformation of the glutathione conjugates of lipid peroxidation-derived aldehydes could also lead to the generation of reactive metabolites that can stimulate mitogenic or inflammatory signaling events. Thus, inhibition of AR could have both salutary and injurious outcomes. Nevertheless, accumulating evidence suggests that inhibition of AR could modify the effects of cardiovascular disease, asthma, neuropathy, sepsis, and cancer; therefore, additional work is required to selectively target AR inhibitors to specific disease states. Despite past challenges, we opine that a more gainful consideration of therapeutic modulation of AR activity awaits clearer identification of the specific role(s) of the AR enzyme in health and disease.

## 1. Introduction

**Figure 1:**
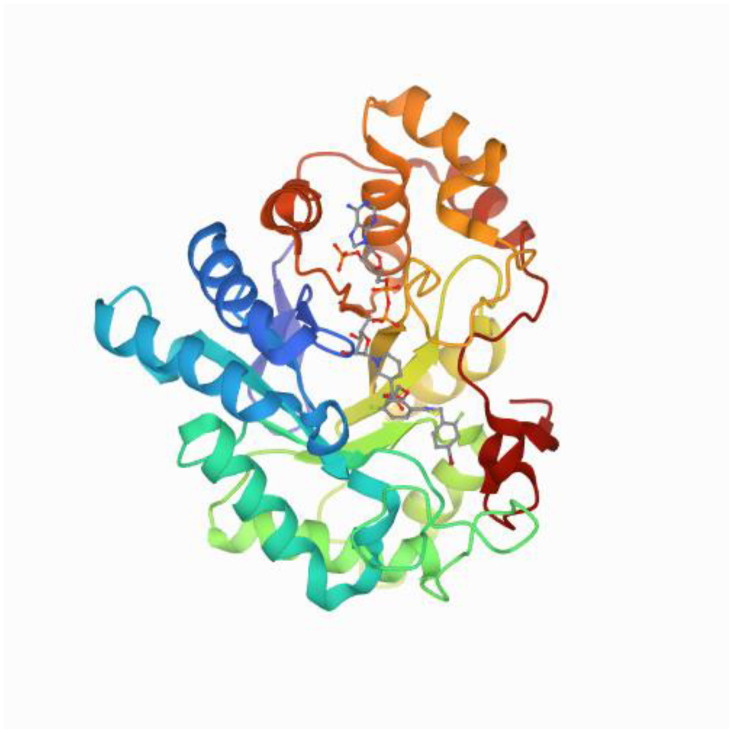
The human aldose reductase (AR). Quantum model of catalysis based on mobile proton revealed by subatomic X-ray and neutron diffraction studies of human aldose reductase (h-AR). The structure presents results of combined studies of the enzyme human aldose reductase (h-AR, 36 kDa) using single-crystal X-ray data (0.66 A, 100 K; 0.80 A, 15 K; 1.75 A, 293 K), neutron Laue data (2.2 A, 293 K), and quantum mechanical modeling. These complementary techniques unveiled the internal organization and mobility of the hydrogen bond network that defines the properties of the catalytic engine, explaining how AR overcomes the simultaneous requirements of efficiency and promiscuity and thus offering a general mechanistic view for this class of enzymes [1], doi:10.2210/pdb2R24/pdb.

**Figure 2:**
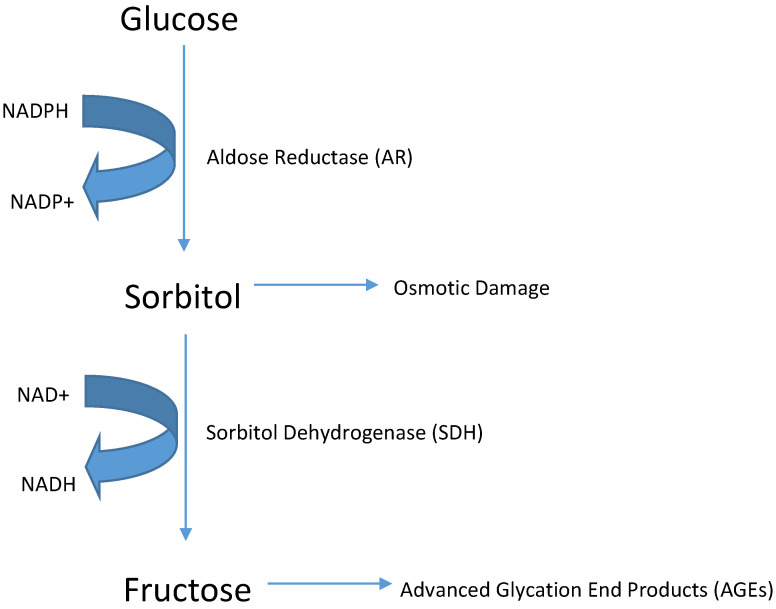
The ‘polyol’ pathway. Products of this pathway are known to affect several vital physiological functions of the cell in the body.

Based on sequence identity, aldo-keto reductases (AKRs) have been grouped into distinct families, AKR1to AKR15; each family contains multiple subfamilies. The AKR1 family has been divided into five subfamilies, A to E. Of these, AKR1B is the most studied subfamily because of the potential role of its founding member, aldose reductase (AKR1B1), in the development of diabetic complications (Figure 1). Aldose reductase (AR) is the first enzyme of the polyol pathway that converts glucose to sorbitol using NADPH as a cofactor. Kinetic properties of AR for glucose, galactose, and various compounds areshown in Table 1. Sorbitol generated by the reaction is converted to fructose by sorbitol dehydrogenase (SDH). These enzymes constitute the ‘polyol pathway’, an alternate route of glucose metabolism, which functions in parallel with glycolysis (Figure 2). 

**Figure 3:**
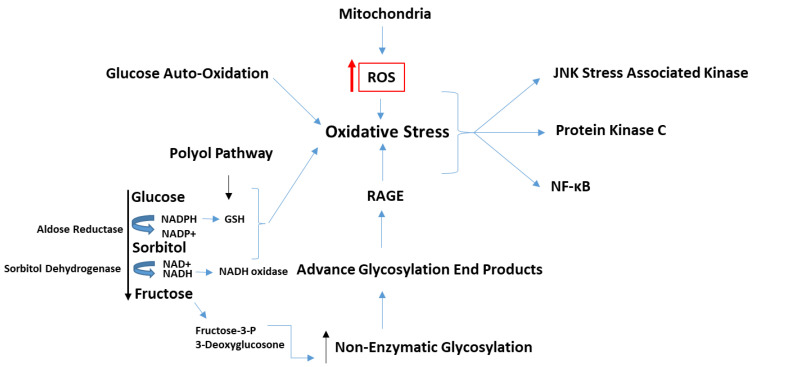
Hyperglycemia induced ‘oxidative-stress’ and its implications. A significant amount of glucose can be shunted via the polyol path, which can lead to oxidative stress through several known signaling pathways affecting vital cellular processes (such as overutilization of NADPH by AR that causes an inability to regenerate GSH and thus decreases the overall antioxidant capacity of the cell). Similarly, SDH results in a buildup of NADH, a substrate for NADH oxidase which produces ROS. Fructose from the polyol path is metabolized to Fructose-3-P, and 3-Deoxyglucasone; both are potent non enzymatic glycation agents. Additionally, glucose auto-oxidation generates H_2_O_2_, O_2_-, and OH^−^, and thus contributes to oxidative stress in cells. Furthermore, binding of AGEs to RAGE is known to generate intracellular stress.

Several studies have demonstrated a role for hyperglycemia in the pathogenesis of diabetic microvascular complications such as retinopathy, nephropathy, neuropathy, and cognitive disorders [2,3]. In addition, the incidence and severity of diabetic micro-vasculopathy are also modulated by the genotype of an individual [4,5]. Although the molecular basis of how hyperglycemia causes tissue injury is still unknown, the mechanism has been linked to what has been termed ‘oxidative stress’ because of the impact of accumulation of sorbitol and advanced glycation end products (AGEs), as outlined in Figure 3. As a putative osmotic regulator under hyperglycemic conditions, AR has been under constant investigation as a critical target to prevent and control diabetic complications, with the view that inhibition of AR could serve as an effective strategy in the prevention or delay of diabetic complications [6]. Although sorbitol is suspected as a main culprit in diabetic complications, the role of sorbitol in this process remains controversial [7,8,9]. Glucose that enters the cell is metabolized in part to sorbitol via AR. AR has very low affinity for glucose, and less than 3% of glucose is processed via this pathway under normal physiological conditions. However, glucose conversion to sorbitol is more pronounced under conditions of chronic hyperglycemia, leading to an accumulation of sorbitol within cells which could increase cell osmolality, and a consequent depletion in intracellular myoinositol. These change, could in turn decrease sodium-potassium adenosine triphosphatase (Na-K-ATPase) activity, and possibly shift the redox potential within the cell. Hyperglycemia could also contribute directly to the decline in cell myoinositol levels by competitively interfering with myoinositol uptake from the extracellular fluid via the sodium-myoinositol cotransporter [8]. If sustained, this chain of events could induce oxidative stress (Figure 3), because high ambient glucose levels increase mitochondrial synthesis of ROS, activating protein kinase C (PKC) and increasing the expression of SDH. 

The generation of superoxide and other ROS are believed to underlie many of the oxidative changes in hyperglycemic conditions. However, despite strong evidence that oxidative stress is associated with diabetic complications, clinical trials with several antioxidants (Table 2) such as (Table 2), α-lipoic acid, vitamins C and E, and growth factors in diabetic neuropathy and retinopathy failed to establish therapeutic efficacies [10], despite strongly positive results (with several exceptions) in rodents [11]. Nonetheless, oxidative stress-induced inflammation is now considered a major contributor to several other disease conditions including sepsis, carcinogenesis and metastasis, allergic asthma, and uveitis as well as the cataract surgery-related posterior capsular opacification. Since ROS-mediated activation of redox-sensitive transcription factors and subsequent expression of inflammatory cytokines, chemokines, and growth factors are characteristics of inflammatory disorders, it was envisioned that by blocking the molecular signals of ROS that activate redox-sensitive transcription factors, various inflammatory diseases could thereby be ameliorated. Indeed, it has been demonstrated that ROS-induced lipid peroxidation-derived lipid aldehydes such as HNE and their glutathione-conjugates (e.g., glutathione-4-hydroxy-trans-2-nonenal, GS-HNE) are efficiently reduced by AR to their corresponding alcohols, which mediate the inflammatory signals [12] (Table 1). These results clearly demonstrated that inhibition of AR significantly prevented the inflammatory signals induced by cytokines, growth factors, endotoxins, high glucose, allergens, and auto-immune reactions in both cellular and in animal models. It was also reported that an AR inhibitor, fidarestat, significantly prevented tumor necrosis factor-alpha (TNF-α), growth factors, lipopolysaccharides (LPS), and environmental allergen-induced inflammatory signals that cause various inflammatory diseases. In animal models of inflammatory diseases such as diabetes, cardiovascular disease, uveitis, asthma, and cancer (colon, breast, prostate, and lung), inhibition of AR significantly ameliorated the disease outcomes. These results from cellular and animal models representing several inflammatory conditions suggest that ROS-induced inflammatory response could be attenuated by inhibiting AR, thereby decreasing disease progression. Because AR inhibitors have already undergone clinical trials for diabetic neuropathy and found to be safe (though clinically not very effective), these observations indicate that they can certainly be developed as potential therapeutics for several inflammation-related diseases in humans [13].

**Table 1:** Aldose reductase (AR) enzyme kinetics. The values for glucose, galactose, and various cytotoxic compounds specific to the AR are shown.

Cytotoxic Compound	Km (mM)	k_cat_ (min^−^^1^)	k_cat_/Km (min^−1^·µM^−1^)	Reference(s)
Acrolein (Figure 4a)	0.80 ± 0.21	37.6	47 M^−1^ min^−1^	[14]
4HNE (Figure 4b)	22	102	4.6 × 10^6^ M^−1^ min^−1^	[15]
Glyoxal (Figure 4c)	514	154	3.0 × 10^5^ M^−1^ min^−1^	[15]
Methylglyoxal (Figure 4d)	0.008	142	1.8 × 10^7^ M^−1^ min^−1^	[15,16]
ONE (Figure 4e)	0.0042 ± 0.0046	92.2 ± 3.55	2190 ± 120 M^−1^ min^−1^	[17]
Glucose (Figure 4f)	0.68	0.15	9.1 × 10^2^ M^−1^ min^−1^	[16,18,19]
Galactose (Figure 4g)	21	222	10.57 M^−1^ min^−1^	[20]

### 1.1. Structure of Aldose Reductase

**Figure 4:**
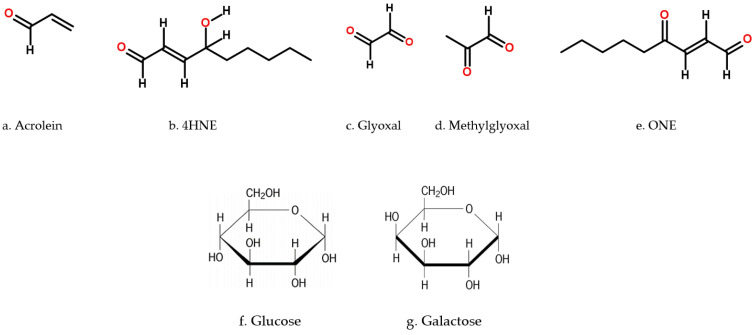
Chemical structure of major substrates metabolized by aldose reductase (AR) enzyme. (**a**). Acrolein (prop-2-enal), (**b**). HNE ((E)-4-hydroxynon-2-enal), (**c**). Glyoxal (oxaldehyde), (**d**). Methylglyoxal (2-oxopropanal), (**e**). ONE ((E)-4-oxonon-2-enal), (**f**). Glucose (6-(hydroxymethyl) oxane-2,3,4,5-tetrol), (**g**). Galactose ((3R,4S,5R,6R) -6-(hydroxymethyl) oxane-2,3,4,5-tetrol).

The NADPH dependent cytosolic AKRs are monomeric enzymes which fold into a typical (α/β)8-barrel structural protein, with a molecular weight ranging from 30–40 kDa [1,2]. Currently, there are more than 150 members distributed in the prokaryotic and eukaryotic kingdoms, including yeasts, plants, invertebrates, and vertebrates; these are expressed in a wide variety of cells and tissues across species [130] (Figure 1). Substrate specificity and inhibitor selectivity are determined by interaction with residues located in three highly variable loops (A, B, and C) (Figure 4). As mentioned earlier, AKR1B1 is related to secondary diabetic complications, while AKR1B10 is induced in cancer cells and is active with all-*trans*-retinaldehyde. Residues that interact with all-*trans*-retinaldehyde and differ between AKR1B1 and AKR1B10 are Leu125Lys and Val131Ala (loop A), Leu301Val, Ser303Gln, and Cys304Ser (loop C). Recently, the importance of Lys125 as a determinant of AKR1B10 specificity for retinoids was demonstrated. Residues 301 and 304 are also involved in interactions with substrates or inhibitors. When members of the rodent AKR1B subfamily AKR1B3 (AR), AKR1B7 (mouse vas deferens protein), AKR1B8 (fibroblast-growth factor 1-regulated protein), and AKR1B9 (Chinese hamster ovary reductase) were tested against all-*trans* isomers of retinaldehyde, and retinol, the enzymes were active with retinaldehyde, but with k_cat_ values (0.02–0.52 min^−1^) much lower than that of AKR1B10 (27 min^−1^) [12]. None of the enzymes showed oxidizing activity with retinol. Since these enzymes (except AKR1B3) have Lys125, other residues may account for retinaldehyde specificity. By using site-directed mutagenesis and molecular modeling to delineate contribution of residues 301 and 304, it was revealed that besides Lys125, Ser304 is a major structural determinant for all-trans-retinaldehyde specificity of AKR1B10 [131].

Renal-specific oxido-reductase (RSOR) has certain structural, and functional similarities to AKR1B, and seems to be relevant to renal complications of diabetes mellitus (DM). Like other AKRs, it has the NADPH binding motif (Figure 1); however, it is located at the N-terminus, and probably undergoes N-linked glycosylation to achieve functional substrate specificity. Besides the AKR3 motif, it has little nucleotide or protein sequence homology with other members of the AKR family. Nevertheless, gene regulation of RSOR, like AKR1B, is modulated by carbonyl, oxidative, and osmotic stress, and thus it is anticipated that it can lead to the development of new inhibitors as well as gene therapy targets to alleviate complications of DM in future [132].

### 1.2. Functional Aspects of Aldose Reductase

Although the focus of research on AR has been its involvement in the development of diabetes, many studies have shown that besides reducing glucose, AR efficiently reduces oxidative stress-generated lipid aldehydes with K_m_ in the micromolar range (10–30 μM). In comparison the Km of the enzyme for glucose is in the millimolar range (50–100 mM) [133,134] (Table 1). There are conflicting reports regarding the actual role(s) played by AR in mammals under physiological and pathophysiological conditions. Past studies suggested a beneficial role for AR in the detoxification of toxic lipid aldehydes generated upon oxidative stress; however, accelerated flux of sorbitol by AR through the polyol pathway (Figure 2) and the subsequent increase in oxidative stress has been strongly implicated in the development of the secondary diabetic complications [135,136,137,138,139,140,141]. Findings that AR inhibitors decrease sorbitol levels and ameliorate complications of diabetes, e.g., cataract in experimental animals strongly support this viewpoint [142]. However, none of the AR inhibitors have passed the phase III clinical trials for the prevention of diabetic complications except epalrestat (sold under the brand name Kinedak^®^ by Ono Pharmaceutical Co., Ltd., Osaka, Japan, now to be manufactured and marketed by Alfresa Pharma Corporation, Osaka, Japan), which has been approved as a pathogenesis-based medicine in patients with diabetic peripheral neuropathy for the improvement of subjective symptoms (numbness and pain), abnormality of vibration sense, and abnormal changes in heartbeat associated with diabetic peripheral neuropathy (in cases of high levels of glycosylated hemoglobin) in Japan [143,144,145]. Recent observations suggest that in addition to increasing sorbitol levels, increased polyol pathway (Figure 3) could potentially alter the NADPH/NADP^+^ ratios and attenuate the glutathione reductase (GR) and glutathione peroxidase (GPx) systems, thereby decreasing the reduced glutathione/oxidized glutathione (GSH/GSSG) ratios, which in turn could cause oxidative stress (Figure 3), one of the major causes of diabetic complications [23,26,27]. This view is supported by studies showing that sugar-induced lens opacification can be significantly prevented by antioxidants such as butylated hydroxytoluene (BHT) and Trolox without further decreasing the elevated levels of sorbitol in the lens [146,147]. Therefore, it seems likely that the effects of inhibiting AR may relate to changes in redox signaling and oxidative stress, rather than sorbitol accumulation per se. 

Our previous studies have shown that lipid peroxidation products such as HNE stimulate rat aortic vascular smooth muscle cell (VSMCs) proliferation that is attenuated by AR inhibitors [148]. We have further demonstrated the mechanistic relationship between oxidant generation, lipid peroxidation, HNE formation, vascular cell cytotoxicity, and vascular complications such as atherosclerosis [141]. The elevated levels of ROS during hyperglycemia, peroxidative stress and cytokine responses are known to trigger inflammation by upregulating redox-sensitive transcription factors such as nuclear factor-kappa B (NF-κB), and activator protein (AP)-1. Modulation of NF-κB has great significance in the mitogenic process mediated by ROS. We also reported that hyperglycemia and TNF-α activate NF-κB and cause proliferation of VSMCs and apoptosis of vascular endothelial cells (VECs) [149,150]. Since hyperglycemia activates NF-κB and cytokines such as TNF-α, which besides activating NF-κB is known to stimulate AR gene expression, it is necessary to further understand the relationship and molecular mechanism underlying these signals. Further, these investigations are important in elucidating the molecular mechanism of inflammatory diseases [13]. 

Currently, AR is widely considered to be an aldehyde-metabolizing enzyme, although reduction of glucose by AR has also been linked to the development of secondary diabetic complications. However, glucose may be an incidental substrate of AR, and the enzyme may be more adept in catalyzing the reduction of a wide range of aldehydes generated from lipid peroxidation (Table 1). In this respect, inhibition of the enzyme has been shown to increase inflammation-induced vascular oxidative stress, and prevent myocardial protection associated with the late phase of ischemic preconditioning [141]. Based on these studies, several investigators have ascribed an important antioxidant role to the enzyme [141,151,152,153]. Ongoing work also indicates that AR is a critical component of intracellular signaling [11,36,37], and inhibition of the enzyme prevents high glucose, cytokine, or growth factor-induced activation of PKC and NF-κB. Thus, treatment with AR inhibitors prevents VSMC growth and endothelial cell apoptosis in culture, inflammation, and restenosis in vivo [141,154,155,156]. Furthermore, studies indicate that the antioxidant, and signaling roles of AR are interlinked, and that AR regulates PKC and NF-κB via a redox-sensitive mechanism. Thus, the available data underscores the need to re-evaluate anti-AR interventions for the treatment of diabetic complications. Potentially, the development of newer drugs that selectively inhibit AR mediated glucose metabolism and signaling without affecting aldehyde detoxification might be useful in preventing inflammation associated with the development of diabetic complications, particularly its micro- and macrovascular implications [141]. 

### 1.3. Role of Aldose Reductase in Human Diseases

#### 1.3.1. Diabetes

Under euglycemic conditions, AR (Figure 2) catalyzes NADP-dependent reduction of glucose to sorbitol, leading to excessive accumulation of intracellular ROS in various tissues of diabetics including heart, vasculature, neuron, eye, and kidney tissues. In vitro cultured cells under high glucose have demonstrated AR dependent increase in ROS production, confirming AR as an important factor for the pathogenesis of diabetic complications [138,152,157,158,159,160]. Based upon extensive experimental evidence showing that the inhibition of AR prevents or delays hyperglycemic injury in several experimental models of diabetes, it has been suggested that AR is one of the main mediators of secondary diabetic complications [135,136,137,138,139,140,141]. Under hyperglycemic conditions, it has been proposed that increased flux of glucose via AR causes osmotic and oxidative stress (Figure 3), which in turn triggers a sequence of metabolic alterations resulting in gross dysfunction in tissues and sub-cellular organelles such as mitochondria, altered intracellular signaling, and extensive cell death [150,161]. The polyol hypothesis centered on the role of AR provided a simple testable paradigm of hyperglycemic injury; however, several key observations regarding diabetic complications are not compatible with the accumulation of sorbitol alone as the major cause of hyperglycemic injury. For instance, in several tissues the intracellular accumulation of sorbitol is not high enough to cause significant osmotic stress [162]. Moreover, the high efficacy of antioxidants in preventing cataractogenesis without preventing sorbitol accumulation suggests that oxidative stress may be an important feature representing hyperglycemic injury [146,147]. In addition to polyol accumulation, other metabolic changes have also been suggested to account for hyperglycemic injury, such as non-enzymatic glycation leading to the accumulation of AGEs and alterations in PKC and myoinositol level [163]. In support of these observations, it was shown that inhibition of PKC or non-enzymic glycation, as well as supplementation with myo-inositol, delayed or prevented hyperglycemic injury. Evidence also suggests that these changes may be interrelated, and that AR might represent a critical link between alterations in PKC, myo-inositol, and non-enzymic glycation [141]. 

Because sorbitol and myo-inositol are structurally similar, depletion of myo-inositol appears to be due in part to the inhibition of its uptake in cells accumulating sorbitol [164]. Furthermore, it was recently demonstrated that stimulation of PKC by phorbol esters up-regulates AR, indicating that some of the effects of PKC activation may be mediated by AR [165]. This finding also suggests that the effects of non-enzymatic glycosylation and AR are inter-related [166,167]. In the past, it was reported that sorbitol-3-phosphate generated by AR is converted to fructose 3-phosphate, which is a better glycosylating agent than glucose, and that AR-mediated catalysis can generate potent glycosylating agents [168]. This view was supported by the observation that in the galactosemic lens (here it is worth mentioning that galactose is a more preferred substrate for AR than glucose since there is a greater accumulation of polyol by the reduction of galactose than glucose, owing to the higher affinity of AR for galactose than for glucose and the fact that there is no subsequent metabolism of galactitol), AR inhibitors suppress the accumulation of advanced Maillard reaction products such as pentosidine, stimulate the actions of aminoguanidine (an inhibitor of non-enzymic glycosylation), and inhibit the accumulation of AGEs [169,170]. Therefore, AR was believed to represent a common mediator of several pathological changes associated with long-term diabetic complications. Further support for a critical role of AR in mediating toxic effects of glucose is provided by acceleration of diabetic cataract by overexpression of AR in the lens of transgenic mice [142]. Previously, we have reported that high glucose in diabetes led to the up-regulation of AR in tissues both in vitro and in vivo, and that treatment with AR inhibitors prevented hyperglycemia-induced hyperplasia, hyperproliferation of VSMCs, and diabetic cardiomyopathy [141,169,171,172,173]. Transgenic overexpression of AR in mice has been shown to exacerbate diabetic cardiomyopathy [174]. These observations indicate that AR inhibitors may be useful in preventing pro-vasculoproliferative effects of diabetes.

#### 1.3.2. Cardiovascular Diseases

Abnormal proliferation of cells of the vascular system is an important feature of atherosclerosis, restenosis, and hypertension. Although several mediators of VSMC growth have been identified, only a few effective pharmacological tools have been developed to limit growth. Evidence indicating an important role for oxidative stress in cellular growth hints at the potential role of AR in the proliferation of VSMCs. Because AR catalyzes the reduction of mitogenic aldehydes derived from lipid peroxidation, it may be a critical regulator of redox changes that accompanies VSMCs growth. Stimulation of human aortic SMCs in culture with mitogenic concentrations of serum, thrombin, basic fibroblast growth factor (bFGF), and HNE led to a 2- to 4-fold increase in the steady state levels of AR mRNA, a 4- to 7-fold increase in AR protein, and a 2- to 3-fold increase in its catalytic activity. Inhibition of AR by sorbinil or tolrestat diminished mitogen-induced DNA synthesis and cellular proliferation. In parallel experiments, the extent of reduction of glutathione conjugate of HNE to glutathionyl-1,4-dihydroxynonene in HNE-exposed VSMCs was decreased by serum starvation or sorbinil. Immunohistochemical staining of cross sections from balloon-injured rat carotid arteries showed increased expression of AR protein associated with the neointima formation. Compared with untreated animals, rats fed with sorbinil displayed 51% and 58% reduction in the ratio of neointima to the media at 10 and 21 days, respectively, after balloon injury [155,173]. Taken together, these findings suggest that AR is upregulated during growth, and that this upregulation facilitates growth by enhancing metabolism of secondary products of ROS [30,31,55,56,58]. 

While it is evident that VSMC proliferation leads to restenosis, mechanisms regulating the growth of SMCs are not known. In one study, it was shown that inhibition of AR could lead to downstream inhibition of NF-κB activation during restenosis in a balloon-injured rat carotid artery, as well as VSMC proliferation due to TNF stimulation. Inhibition of VSMC growth by AR inhibitors was not accompanied by increase in cell death or apoptosis. Inhibition of AR led to decrease in the activity of transcription factor NF-κB in culture, and in the neointima of rat carotid arteries after balloon injury. Inhibition of AR in VSMCs also prevented activation of NF-κB by bFGF, angiotensin-II (Ang-II), and platelet-derived growth factor (PDGF-AB). The VSMCs treated with AR inhibitors showed decreased nuclear translocation of NF-κB and diminished phosphorylation and proteolytic degradation of IκB-α. Under identical conditions, treatment with AR inhibitors also prevented the activation of PKC by TNF-α, bFGF, Ang-II, and PDGF-AB, but not phorbol esters, indicating that AR inhibitors prevent PKC stimulation or availability of its activator, but not the PKC itself. Treatment with antisense AR, which decreased the AR activity by >80%, attenuated PKC activation in TNF-α, bFGF, Ang-II, and PDGF-AB-stimulated VSMCs, and prevented TNF-α induced proliferation. Results from this study suggest that inhibition of NF-κB may be a significant cause of the anti-mitogenic effects of AR inhibition, and that this may be related to disruption of PKC associated signaling in the AR inhibited cells [172]. 

In atherosclerosis, vascular lesions are commonly associated with the accumulation of oxidized lipids (products of lipid oxidation), particularly aldehydes (Table 1). These lipids stimulate cytokine production and enhance monocyte adhesion; however, their contribution to atherosclerotic lesion formation is not yet clear. Examination of atherosclerotic lesions in apolipoprotein (apo)E-null mice revealed that AR was localized in macrophage-rich regions, and that its abundance increased with lesion progression. Treatment of apoE-null mice with AR inhibitor sorbinil or tolrestat increased early lesion formation but did not affect the formation of advanced lesions. Early lesions in AR (−/−)/apoE (−/−) mice maintained on high-fat diet were significantly larger when compared with age-matched AR (+/+)/apoE (−/−) mice. The increase in lesion area attributable to deletion of the AR gene was seen in both genders of mice. Pharmacological inhibition or genetic ablation of AR also increased the lesion formation in male mice made diabetic by streptozotocin (STZ) treatment. Lesions in AR (−/−)/apoE (−/−) mice exhibited increased collagen and macrophage contents and a decrease in SMCs. AR (−/−)/apoE (−/−) mice also displayed a greater accumulation of the AR substrate HNE in the plasma and protein-HNE adducts in arterial lesions than AR (+/+)/apoE (−/−) mice (Figure 4). These observations indicate that AR is upregulated in atherosclerotic lesions, and that it protects against early stages of atherogenesis by removing toxic aldehydes generated in oxidized lipids [175]. 

When cultured in a high level of glucose but not iso-osmotic mannitol, VSMCs increase membrane-associated PKC activity. This was prevented by tolrestat or sorbinil, or by ablation of AR using small interfering RNA (siRNA) [134]. Tolrestat also prevented phosphorylation of PKC β2 and δ isoforms. Other experiments in VSCMs revealed that inhibition of AR, independent of SDH, reduced both oxidative stress and hyperglycemic changes in signaling upstream to the activation of multiple PKC isoforms and PLC-γ1, suggesting that such therapy might prevent secondary vascular complications of diabetes [152,171,176,177,178]. Both AR inhibitors and vasodilators improved nerve conduction velocity in STZ diabetic rats, suggesting a metabolic vascular interaction that could be mediated by restoration of impaired nitric oxide synthesis [179]. Daily administration of epalrestat, an AR inhibitor, to STZ diabetic rats prevented gastric erosion and ulceration and normalized gastric mucosal blood flow, an effect also found with a nitric oxide synthase inhibitor [180]. These results suggest that AR inhibitors may act at least in part by blocking the action of induced nitric oxide. However, AR inhibitors did not prevent or improve retinopathy in alloxan-induced diabetic dogs [181]. In one study, in which alloxan-induced diabetic dogs were treated for five years, there was no evidence of reduced capillary basement membrane thickening in the retina, renal glomerulus, or leg muscle [182].

Type 2 diabetes is associated with platelet hyperactivity, which leads to increased morbidity and mortality from cardiovascular diseases. This is coupled with enhanced levels of thromboxane (TX), an eicosanoid that facilitates platelet aggregation. The mechanism underlying the relationship among hyperglycemia, TX generation, and platelet hyperactivity remains at large. In human platelets, AR synergistically modulates platelet response to both hyperglycemia and collagen exposure through a pathway involving ROS/PLCγ2/PKC/p38α MAPK. In patients with platelet activation (i.e., deep vein thrombosis, saphenous vein graft occlusion after coronary bypass surgery), and particularly in those with diabetes, urinary levels of a major enzymatic metabolite of TX (11-dehydro-TXB2 [TX-M]) have been shown to be substantially increased [183]. Thus, in this setting, AR pathway-mediated oxidative stress can lead to enhanced platelet TX generation or thromboxane receptor activation. 

### 1.4. Asthma 

It has been reported that ROS and ROS-derived lipid peroxidation products like HNE and malondialdehyde (MDA) are involved in asthma pathogenesis, and that the reduced products of GS-lipid aldehydes such as GS-lipid-alcohols are important inducers of signaling cascades. AR inhibition has been shown to interfere in the formation of GS-lipid alcohol species as ROS-mediated activation of inflammatory signals that are blocked by AR inhibition [184]. Also, studies in mice indicate that AR inhibitors could serve as potential anti-inflammatory interventions. When sensitized mice were challenged with ragweed pollen extract or the carrier (without ragweed pollen extract), robust airway inflammation was observed. Mice treated with the AR inhibitor sorbinil revealed significantly less inflammation as determined by the number of eosinophils in bronchoalveolar lavage fluid. Similarly, perivascular and peri-bronchial inflammation and cell composition in the bronchoalveolar lavage fluid induced by ragweed pollen extract challenge was significantly prevented by sorbinil. Further, AR inhibition also prevented ragweed-induced mucin production and airway hyperresponsiveness in mice after methacholine challenge [185]. These results indicate that AR inhibition significantly prevented the pathophysiological effects of a common natural allergen, ragweed pollen extract-induced asthma in a murine model. Effectiveness of AR inhibition was also examined in acute ovalbumin (OVA) induced airway inflammation in mice. In fact, studies with OVA-challenged chronic asthmatic mice showed a decrease in total as well as differential counts with the addition of an AR inhibitor. In the same study, it was noted that airway hyper-responsiveness measured by whole body plethysmography was reduced by the addition of fidarestat. The OVA-induced mice had a significant decrease in Penh times, which is a measure of changes in breathing established through whole body plethysmography. Further, TGF1β1 released from damaged or repairing epithelium is a mediator of airway remodeling. AR inhibition was shown to prevent the effect of TGF1β1 on epithelial mesenchymal transition through a non-canonical Smad independent pathway [186].

A detailed examination of OVA sensitization and challenge showed a clear and marked perivascular and peri-bronchial infiltration of eosinophils into the lungs of the mice. Such infiltration of inflammatory cells into the airways of OVA-challenged mice was reduced with an AR inhibitor. More importantly, protective effect of the AR inhibitor against OVA-induced airway inflammation coincided with a significant reduction in the levels of Th2 cytokines, including IL-4, IL-5, and IL-6 and chemokines such as keratinocyte-derived chemokine, as well as granulocyte colony stimulating factor (G-CSF) and MCP-1 in broncho-alveolar lavage fluid [187], indicating a novel role for AR inhibitors in the prevention of asthma [13]. 

### 1.5. Oculopathy

Diabetic retinopathy (DR) is one of the common microvascular disorders, and the most severe of diabetic ocular complications. DR has been shown to be significantly associated with impending risk of cerebrovascular accident (CVA), myocardial infarction (MI), congestive heart failure (CHF), and all-cause mortality in patients with type 2 diabetes mellitus, suggesting that patients exhibiting higher degrees of retinopathy appear to carry a heightened risk for each of the above mentioned outcomes; thus retinal information may provide valuable insights into patients’ risk of future vascular disease including death [188]. In the past, special attention has been paid to the role of AR in its pathogenesis. Several associations between AR and early diabetic retinopathy have been described in detail, including the localization of AR in the retina; the role for increased AR activity in retinal capillary cell loss and formation of acellular capillaries, capillary basement membrane thickening, increased vascular permeability, and disruption of the blood-retinal barrier; increased leukocyte adhesion to endothelial cells; neovascularization with advanced proliferative DR (as learnt from animal models of diabetes); and galactose feeding [189,190,191,192]. Further, potential mechanisms underlying the interactions between AR and other pathogenetic factors such as formation of AGEs; oxidative-nitrosative stress; PKC, MAPK, and poly (ADP-ribose) polymerase activation; inflammation; and growth factor imbalance have been studied [193]. AR inhibitors have been found to prevent diabetic cataract formation in almost all animal models tested so far. It is believed that AR inhibition reduces oxidative stress and retinal neovascularization. In a mouse model of oxygen-induced retinopathy (OIR), seven-day-old normal and AR-deficient mice were exposed to 75% oxygen for five days and then returned to normal room air. Parameters such as vascular obliteration, neovascularization, and blood vessel leakage were analyzed. In comparison to normal OIR retinae, AR deficient OIR retinae displayed significantly smaller central retinal vaso-obliterated area, less neovascularization, and reduced blood vessel leakage. There was also a significant reduction in oxidative stress and glial response in the AR deficient OIR retinae. Moreover, reduced microglial response in the avascular area with increased microglial responses in the neovascular area were found with AR deficiency. Interestingly, expression levels of VEGF, p-Erk, p-Akt, and p-IκB were significantly reduced in AR deficient OIR retinae, indicating that AR deficiency reduced retinal vascular changes in a mouse model of OIR, and that AR can be a potential therapeutic target in ischemia-induced retinopathies [194].

In another study, in vivo evaluation of the potency of fidarestat in STZ treated diabetic rats was conducted for diabetes-associated cataract formation, retinal oxidative nitrosative stress levels, glial activation, and apoptosis. Fidarestat treatment prevented diabetic cataract formation and successfully counteracted retinal nitrosative stress and poly (ADP-ribose) polymerase activation as well as glial activation. It also prevented nitrotyrosine, poly (ADP-ribose) accumulation, and apoptosis. These findings supported an important role for AR in diabetes-associated cataract formation, as well as retinal oxidative-nitrosative stress, glial activation, and apoptosis. These results provide a strong rationale for the development of AR inhibitors such as fidarestat for the prevention and treatment of diabetic ocular complications [195]. 

Paradoxically, not all patients with diabetes develop ocular complications. Some diabetics with poor metabolic control appear to be protected against retinopathy, while others with a history of excellent metabolic control develop severe complications. These observations indicate that some risk factor(s) may influence the likelihood that an individual with diabetes will develop cataract and/or retinopathy. It seems that an elevated level of AR gene expression could confer higher risk for the development of diabetic eye disease. A transgenic mouse strain harboring human AR was examined for the onset and severity of diabetes-induced cataract. AR-TG mice homozygous for the transgene demonstrated a conditional cataract phenotype whereby they developed lens vacuoles and cataract-associated structural changes only after induction of experimental diabetes; no such changes were observed in AR-TG heterozygotes or non-transgenic mice with or without experimental diabetes induction. The nondiabetic AR-TG mice did not show structural changes in the lens even though they had lenticular sorbitol levels almost as high as the diabetic AR-TG lenses that showed early signs of cataract. However, overexpression of AR led to increase in the ratio of activated to total levels of extracellular signal-regulated kinase (ERK1/2) and c-Jun N-terminal (JNK1/2), which are known to be involved in cell growth and apoptosis, respectively [10,38,80]. After diabetes induction, AR-TG, but not WT controls, had decreased levels of phosphorylated as well as total ERK1/2 and JNK1/2 compared to their nondiabetic counterparts. These results indicate that high AR expression in the context of hyperglycemia and insulin deficiency may constitute a significant risk factor that could predispose the lens to disturbance in signaling through the ERK and JNK pathways, and thereby alter the balance of cell growth and apoptosis that is critical to lens transparency and homeostasis [196]. However, the general experience with AR inhibitors in a randomized double-blind study of patients with insulin-dependent diabetes (IDDM), either with sorbinil or placebo, was found to be disappointing as no significant differences were noted between the treatment and placebo groups. Unfortunately, hypersensitivity to sorbinil was noted in about seven percent of subjects during the treatment. Ponalrestat, tested in small groups of patients, also showed no benefit in DR [197], nor did a trial of topical corneal administration of an AR inhibitor [198].

More recently, advances in molecular genetics are enabling studies to discern the influence of individual genes among patients with similar risk factors for the development and progression of DR because of the substantial variability in the progression of disease and its severity. Polymorphism in the promoter of the AR gene has been suggested in changing the expression of its gene in certain ethnic groups. In a limited number of type 2 diabetic patients categorized for the presence or absence of diabetic microangiopathy, AR genotyping revealed a C-106T polymorphism that turned out to be a risk factor for the development of retinal complications in those patients [199]. A few of them have shown statistically significant association in multiple series from various parts of the world; however, no definite genetic association with DR has been consistently reported. This lack of association could be due to small sample size, study design limitations, underlying genetic differences between study populations, or other unknown factors. 

Apart from its association with the etiology of DR, AR has also been linked to the causation of autoimmune-mediated uveitis. Earlier studies suggested that inhibition of AR prevented cytokine, growth factor, and LPS-induced oxidative stress signals, leading to production of prostaglandin E (PGE) 2, cytokines, and activation of cycloxygenase-2 (Cox-2) and iNOS; activation of these inflammatory markers is known to be the major mediator of ocular inflammation. Srivastava and colleagues investigated the effect of AR inhibition on endotoxin-induced uveitis (EIU) in rats [200]. Inhibition of AR prevented EIU-induced inflammatory marker levels in the aqueous humor (AQH) of rat eyes and suppressed the inflammatory cells’ (leukocytes) infiltration and protein concentration in AQH. Similarly, the rise in TNF-α, nitric oxide (NO), and PGE2 levels in AQH of EIU rats was significantly attenuated by AR inhibition. Furthermore, the levels of TNF-α in the anterior and posterior segments of the eye were also determined. Increased levels of TNF-α were found in AQH, vitreous humor, choroid, and retina, and were significantly prevented by AR inhibition. Similarly, the increase in iNOS and Cox-2 proteins in the ciliary body, corneal epithelium, and retinal wall were prevented by AR inhibition.

Administration of AR inhibitor also prevented the activation of NF-kB in the ciliary body, corneal epithelium and retinal wall of LPS-treated rat eyes, suggesting that inhibition of AR prevents EIU in rats [200]. Thus, AR inhibitors could be employed therapeutically to treat patients with uveitis and associated complications that have the potential of stimulating the inflammatory signals [13]. The mechanistic details of how AR regulates the redox signaling are not clear; however, the evidence collected in our laboratory indicates that oxidative stress (Figure 3) generates large number of lipid-derived aldehydes by peroxidation of membrane lipids, which readily conjugate with glutathione and are reduced to respective alcohols by AR (Table 1). The reduced GS-lipid alcohols act as signaling intermediaries and activate protein kinases via a still uncertain mechanism, eventually activating redox-sensitive transcription factors, causing inflammation and further enhancement of the prevailing oxidative stress and continuing cyclic episodes that lead to disease establishment and progression [141,201]. Interestingly, inhibition of AR blocks the production of GS-lipid alcohols which could halt this cycle and prevent disease progression [202].

### 1.6. Nephropathy

Diabetic nephropathy (DN) is the principal cause of end-stage renal disease in Western society, affecting a substantial proportion (25–40%) of patients [203]. The pathogenesis of DN involves hemodynamic changes that include elevation of systemic and intraglomerular pressure and activation of vasoactive hormonal pathway including the RAS, endothelin, and urotensin. One of the primary factors in the development of DN is the mesangial extra cellular matrix (ECM) accumulation. Studies show that AR inhibitors (Table 2) decrease the mesangial matrix in diabetic rats by 80–90% via inhibition of TGF-β [204,205,206]. 

In the past, treatment of DN has focused on controlling the hyperglycemia and interrupting RAS with anti-hypertensive agents. Novel targets, some of which are linked to glucose dependent pathways targeting the AR, appear to be a major focus of new therapies directed against the development and progression of renal damage. It is likely that resolution of DN will require synergistic therapies to target multiple mediators [207]. Genetic association with AR and susceptibility to diabetic microvascular complications have been described by several groups. Preliminary reports associate a tendency to nephropathy in diabetics having polymorphism of the gene coding for AR in both type 1 [208] and type 2 [209] diabetics. Additionally, polymorphism in the promoter region of the gene, the gene itself, and in other regions have also been shown to be correlated with diabetic complications including nephropathy [210]. One polymorphism is (AC)n dinucleotide repeat at the 5′ end of the AR gene. The Z-2 variant is associated with a 2- to 3-fold increase in the expression of AR in humans with diabetic nephropathy. Expression of this genotype exhibited a 3.3-fold increase for the risk of classic diabetic glomerulopathy [211]. 

A meta-analysis of studies on this association concluded that Z-2 allele appeared to be a genetic risk factor for DN [212]. ARIs may improve some of the manifestations of DN, since experimental models have shown a decrease in hyperperfusion during DN with the use of ARIs (Table 2). Diabetic mice treated with sorbinil showed normal single nephron filtration rate, plasma flow, and blood flow whereas the markers for glomerular hyperperfusion were all raised in the untreated mice [213]. Further, in a study using three different ARIs all reduced the glomerular filtration rate and the 24 h urinary albumin excretion when compared to untreated diabetic mice [214]. In one study, for example, when tolrestat was given to patients with IDDM after six months of placebo [215] it reversed the glomerular hyperfiltration, lowering the glomerular filtration rate from 156 to 124 mL/min and decreasing urinary albumin excretion rate from 197 to 158 mg/day. Similar changes were observed with ponalrestat treatment [216].

It has been hypothesized that ARI treatment lowers plasma VEGF in diabetic rats, and this may contribute to ARIs’ anti-microalbuminuric effects [217]. Despite previous evidence, some studies suggested that ARs may not be effective over the long term. In one report, urinary albumin excretion was measured in diabetic subjects who were treated either with tolrestat or ascorbic acid [218]. Although ascorbic acid decreased urinary albumin excretion rate after nine months, tolrestat had no effect on proteinuria or other measured variables. On the other hand, when epalrestat was evaluated in a study that administered the drug to type 2 diabetics presenting with microalbuminuria and who were then compared with age, gender, and body mass matched diabetic control subjects [219] blood pressure, HbA1c, and total cholesterol were unchanged in both groups, and the control group had significantly increased urinary albumin excretion from 82 mg/g creatinine at the baseline to 301 mg/g at the end of the study. There was no change in urinary albumin excretion in the epalrestat treated group (81 mg/g at baseline and 87 mg/g by the end of the study). However, a further illustration of the benefits of ARIs in DN is that increased AR expression, AGE, and TGF-β were inhibited by zopolrestat [220]. These results were interpreted by the investigators as favoring the potential usefulness of ARIs in the incipient stage of DN in T2D.

### 1.7. Neuropathy

Diabetic peripheral neuropathy (DPN) is a common yet painful and severely debilitating complication that currently has no effective treatments other than diabetes control and management. The recognition of the difficulty in reversing established DPN has focused efforts primarily on slowing its progression [221]. ARIs have produced inconsistent benefits in DPN. A double-blind placebo-controlled trial evaluated the effect of tolrestat withdrawal in patients with IDDM and symptomatic neuropathy who were treated with tolrestat [222]. Patients who were switched to placebo showed progression of motor nerve neuropathy (decreased conduction velocity) and deterioration of vibration threshold. In comparison, those who continued tolrestat remained stable. Symptoms of pain and paresthesia were less prevalent in the tolrestat group. Some benefits of tolrestat in primary prevention [223] and in the treatment of symptomatic diabetic neuropathy have been shown [66,122,224,225]. However, the improvements were relatively minor, and only seen in a relatively small number of symptomatic patients [7].

In one study of patients with symptomatic neuropathy, a sustained improvement in motor nerve conduction velocity and paresthesia was seen in 28 percent of patients treated with tolrestat, versus five percent in the placebo group. There was no benefit in neuropathic pain, which improved to an equivalent degree in both groups. A multicenter double-blind trial involving 549 patients with sensorimotor polyneuropathy who were assigned to treatment with placebo or ranirestat 10, 20, or 40 mg/day for 52 weeks found no significant change in nerve conduction studies, or in quantitative sensory tests of bilateral sural plus proximal median sensory nerve conduction velocity [101]. There was significant improvement in the summed motor velocity with ranirestat 20 and 40 mg/day at 12, 24, and 36 weeks, and in peroneal motor velocity with ranirestat 20 mg/day at 36 and 52 weeks. The authors concluded that treatment with ranirestat benefits motor but not sensory nerve function in mild to moderate neuropathy [226]. Based on these data, ranirestat was termed as the ‘most promising’, and ‘safe’ of the newly introduced ARIs (Table 2) [101].

At present, there is decreased enthusiasm for ARIs to prevent or treat diabetic complications, since the large sorbinil retinopathy trial showed a lack of important benefits [44]. Although nerve conduction velocities were increased by sorbinil in the peroneal nerve they were not in the median motor or sensory nerves as there was no amelioration of the early clinical signs or symptoms of diabetic neuropathy [45]. A similar lack of benefit was observed in a second study, in which an autonomic neuropathy was assessed using myocardial scintigraphy [227]. Despite setbacks, encouraging studies in rats prompted clinical trials of fidarestat. By donating a proton to AR, fidarestat changes from a neutral to a negative charge, a change that is thought to underlie the mechanism for its inhibitory action [228]. Control and STZ-induced diabetic rats were treated with or without fidarestat for six weeks after induction of diabetes. Sciatic motor nerve conduction velocity, hind limb digital sensory nerve conduction velocity, and sciatic nerve concentration of two major non-enzymatic antioxidants, glutathione, and ascorbate, decreased in diabetic versus control rats, and these changes were prevented by fidarestat. In addition, fidarestat prevented diabetes-induced increase in nitrotyrosine (a marker of peroxynitrite-induced injury), and poly (ADP-ribose) immuno-reactivities in both the sciatic nerve and retina [229]. In a study of 279 patients with diabetic neuropathy, patients were randomly assigned to receive placebo or fidarestat for 52 weeks [52]. Significant objective improvement in electrophysiological measures of nerve conduction in motor, and sensory nerves was observed in the fidarestat group compared with placebo, and all subjective symptoms evaluated (numbness, spontaneous pain, sensation of rigidity, paresthesia in the soles upon walking, heaviness in the foot, and hypesthesia) were also significantly improved in the treated group. However, no other clinical trials involving fidarestat have been reported since 2005.

One positive clinical application has been reported in a Japanese trial of the AR inhibitor epalrestat [230,231]. In a prospective open-label multicenter study in which epalrestat and control groups were randomly assigned to either epalrestat daily (289 patients) or a control group (305 patients) for three years, with median nerve conduction velocity (MNCV) taken as the key determinant of efficacy, Epalrestat significantly prevented deterioration of median MNCV while preserving vibration perception threshold seen in the control group. Long-term treatment with epalrestat was well tolerated. 

Clinical end points for improvement in diabetic neuropathy are difficult to quantitate, and caution is appropriate in accepting a positive conclusion from those studies reported to date [7]. Except for fidarestat, there has generally been no improvement in pain, an inconsistent effect on paresthesia, and an increase in nerve conduction in some but not all nerves. To provide more objective data, sural nerve biopsies were examined in a trial of tolrestat in 600 diabetic subjects in the hope that histologic evidence of nerve regeneration may be a marker of drug induced improvement [232]. Ranirestat was found to penetrate the sural nerve and inhibit sorbitol and fructose accumulation in patients with diabetic sensorimotor polyneuropathy [99]. However, overall clinical trials to demonstrate prevention or amelioration of diabetic neuropathy by ARIs have proven insufficiently positive to warrant their clinical use. Current exploration of the benefits of blocking AR in diabetic patients should include correction of flawed clinical trial design, introduction of improved drugs, and exploration of how the molecular anatomy of an AR drug relates to its efficacy [233]. Interestingly, in a diabetic AR knockout mice study a delayed onset of nerve conduction slowing was observed in comparison to that of diabetic wild-type (WT) mice. Further, when sciatic nerves from these mice were exposed to 12 weeks of diabetes followed by a metabolomics analysis, it led to an identification of elevated glucosamine levels in both diabetic AR knockout as well as diabetic WT mice, identifying a novel pathway. Thus, exploration of new pathway(s) may offer a potential therapeutic target in diabetic neuropathy [234].

### 1.8. Sepsis

Although AR was initially studied for its role in the pathogenesis of diabetic complications, but it has also gained attention for its role in several inflammatory conditions [233]. One of these conditions is sepsis, a fatal inflammatory response syndrome which develops when the initial host response to microbes or microbial products is amplified uncontrollably. Studies suggest that inhibition of AR can attenuate the inflammatory signaling involved in the production of cytokines and chemokines during sepsis. Furthermore, inhibition of AR has been shown to decrease cytotoxic effects associated with inflammatory mediators in tissues, as well as their paracrine and endocrine effects that propagate toxicity [13]. 

Lipid peroxidation derived carbonyls generated by oxidative stress are catalyzed to their glutathione adducts by AR [12]. These adducts, including GS-HNE, propagate the inflammatory signals through the activation of PLC/PKC/NF-κB, and findings show that the harmful effects of uncontrolled inflammation can be effectively prevented or significantly ameliorated by inhibiting AR, either with a pharmacological AR inhibitor or by genetic ablation of AR through small interfering RNA (siRNA) [140]. Previous work showed that treatment of LPS stimulated macrophages with AR inhibitors prevented the activation of NF-κB and other pro-inflammatory markers such as NO, PGE2 and COX-2 [235]. A study looking at LPS-induced murine peritoneal macrophages showed that treatment with three different ARIs significantly reduced (80–90%) levels of TNF-α, IFN-γ, IL-1β and MCP-1 (Table 2).

Moreover, it has been shown that inhibition of AR prevents LPS-induced secretion of cytokines in serum, liver, kidney, spleen, and heart along with a concomitant decrease in mouse cardiac muscle contractility that leads to cardiomyopathy and lethality in a mouse model of sepsis [236]. Similarly, AR inhibition also prevents cecum ligation puncture-induced inflammatory response in mice [237]. All these studies suggest that AR inhibitors that are thought to prevent some diabetic complications could also be alternatively employed to downregulate septic cascade, and its associated lethality.

### 1.9. Cancer

Recent studies suggest that AR is a key regulator of ROS signals induced by cytokines and growth factors (GF) leading to cell growth and differentiation in vascular cells and colorectal cancer (CRC) cells [55,127,128]. RNA interference ablation of AR or pharmacological inhibitors of AR prevent growth factor and cytokine-induced cancer cell growth. Further, inhibition of AR by sorbinil or by antisense ablation prevented FGF and PDGF induced upregulation of PGE2 synthesis in Caco-2 cells. Inhibition of AR also prevented GF induced Cox-2 activity, protein, and mRNA, and significantly decreased the activation of NF-κB, PKC, and phosphorylation of PKC-β2, as well as progression of Caco-2 cell growth, but had no effect on Cox-1 activity. Cell cycle analysis suggested that inhibition of AR prevented GF-induced proliferation of Caco-2 cells in S-phase. Since ROS are major culprits in the uncontrolled growth of cancer cells, researchers also examined the effect of AR inhibition on ROS production. These results suggest that AR inhibition prevents GF induced ROS production in cancer cells [238]. Further, it has also been shown that inhibition of AR prevents cancer cell growth by suppressing the entry of cells in the G1/S phase of the cell cycle via regulating the transcriptional activation of E2F transcription factors. The efficacy of ARI or AR siRNA in the prevention of colon cancer growth in nude mice xenografts was also examined. Inhibition or siRNA ablation of AR completely halted the growth of human adenocarcinoma cells (SW480) in nude mice xenograft tumors [239]. None of the treatments interfered with the normal weight gain of animals during the experiments.

AR inhibition prevented the azoxymethane (AOM)-induced aberrant crypt foci (ACF) formation and premalignant lesions in mice. These studies indicated that AR null mice are resistant to AOM-induced ACF formation, and expression of inflammatory and carcinogenic markers [240,241]. Several studies indicate that AR is overexpressed in human cancers such as lung, colon, breast, and prostate [242]. Further, AR is overexpressed in hepatocarcinogenesis [243]. Previous studies also indicate that AR inhibition prevents colon cancer cachexia [82]. The above studies thus indicate that inhibition of AR may be a potential therapeutic approach in preventing progression of CRC. Additionally, AR inhibition suppresses the oncogenic miR-21 expression and prevents proliferation of cancer cells. PTEN as a putative target of miR-21 was identified, and it was shown that AR inhibition upregulates PTEN and FOXO3a levels, and downregulates miR-21, thereby facilitating the inhibition of tumorigenesis. It was demonstrated that AR regulates the expression of miR-21 and its target PTEN, which are the key regulators of numerous physiological cellular processes such as proliferation, metabolism, and apoptosis via PI3K/AKT/AP-1 signaling [244].

## 2. Concluding Remarks, and Perspectives

AR is an important enzyme that has been the focus of active research for more than four decades. It is a rate-limiting enzyme in the polyol pathway and has been consistently implicated in the pathogenesis of diabetic complications. The enzyme catalyzes the reduction of glucose to sorbitol. So far, outcomes from clinical studies targeting its inhibition have not led to robust and convincing evidence of significant benefits, except in some patients with diabetic neuropathy. It is possible that doses employed for various ARIs were low (in anticipation of possible toxicity at higher doses), or that studies were not done for long enough (Table 2) [7]. Microvascular complications of diabetes develop slowly, often taking a decade or longer to become clinically evident. Therefore, an intervention in the late stages of nephropathy, neuropathy, or retinopathy may not result in significant differences between treatment groups. The inability of ARIs to reproduce clinical benefits observed in animal models in diabetic patients by blunting neuropathy and retinopathy has been disappointing. Thus, although one in four patients with diabetes is afflicted with distal symmetric polyneuropathy, treatment of the syndrome continues to be an unmet challenge [137].

As the molecular basis for the pathogenesis of diabetic complications is further unraveled, an increasingly promising therapy would be to administer two or more synergistic metabolic blockers. As an example, expression of retinal VEGF and development of proliferative retinopathy in galactosemic rats may be prevented by either aminoguanidine or ARIs. Since both drugs impede immunocytochemical expression of VGEF, their combined therapy may ultimately prevent diabetic retinopathy [137,245]. The role of AR in mediating angiogenic signals has been examined in cell culture and in nude mice xenografts [156]. In one study, the inhibition of AR prevented VEGF-induced proliferation and expression of oxidative stress signaling, infiltration of blood cells, invasion, migration, and formation of capillary-like structures, suggesting a possible application in preventing angiogenesis. For now, clinical application of ARIs remains an unfulfilled promise.

Although AR was initially thought to be involved in secondary diabetic complications because of its glucose reducing potential, evidence from recent studies indicates that AR is an excellent catalyst for the reduction of a number of lipid peroxidation-derived aldehydes as well as their glutathione conjugates, which regulate inflammatory signals initiated by oxidants such as cytokines, growth factors, and bacterial endotoxins, and thus revealing the potential use of AR inhibition as an approach to prevent inflammatory complication (Table 1). Nonetheless, inhibition of AR appears to be a promising strategy for the treatment of endotoxemia, sepsis, and inflammatory diseases. Current knowledge provides enough evidence to indicate that AR inhibition is a logical therapeutic strategy for the treatment of endotoxin related inflammatory diseases. Since ARIs have already undergone clinical studies for diabetic complications and found to be safe for human use, their use in endotoxin related inflammatory diseases could be expedited (Table 2). However, one of the major challenges will be the discovery of AR-regulated clinically relevant biomarkers to identify susceptible individuals at risk of developing inflammatory diseases, thereby warranting future research [246]. 

There are obvious challenges associated with in vivo studies, as AR-like enzymes such as AKR1B10, AKR1B7, and AKR1B8 are highly related isoforms and share up to 65% protein identity, and are often co-expressed along with AR, making functional analysis a difficult task. They can reduce many redundant substrates (aldehydes from lipid peroxidation, steroids and their byproducts, and xenobiotics, in vitro) (Figure 4). Based on these properties it appears that more holistic approaches may be designed to study the AKR family, which are involved not only in detoxification but signal transduction as well. Therefore, inhibition of these enzymes might have unforeseen physiological consequences [130]. 

Apart from its role as a detoxifying enzyme of toxic aldehydes (Table 1), AR is also an osmoregulator in the kidney and a regulator of sperm maturation. Emerging reports now suggest that under normal glucose concentration AR may be upregulated by factors other than hyperglycemia and be involved in other pathological processes that have become major threats to human health. Such pathologies include cardiac disorders, inflammation, mood disorders, renal insufficiency, and ovarian abnormalities [43,209,247,248,249,250,251,252,253,254,255,256,257,258]. In addition, AR is overexpressed in different human cancers such as liver, breast, ovarian, cervical, and rectal cancers, suggesting that AR may be an attractive target for anti-cancer interventions [242,247,251,259,260,261,262,263,264,265,266]. 

Past research indicates that AR is involved in the pathogenesis of secondary diabetic complications. As a result, several ARIs were developed and tested in the setting of diabetic complications (Table 2). These inhibitors were found to be safe for human use but were found to be unsuccessful in clinical studies because of their limited efficacy. Looking at the trend of the last few years, it appears that the research for new chemical entities has relatively subsided; however, natural products and plant extracts with AR-inhibitory activities are gaining more interest, along with the search for proper forms of known inhibitors that are based on a rational receptor-focused lead optimization endowed with lower micromolar or sub-micromolar activities as a way to improve their impaired physicochemical profiles, as well as potential combination therapies with other compounds of pharmaceutical interest [267,268]. 

Recently, several groups have suggested that besides reducing glucose, AR also efficiently reduces oxidative stress-generated lipid peroxidation derived-aldehydes and their glutathione conjugates. Since lipid aldehydes alter cellular signals [12] by regulating activation of transcription factors such as NF-κB and AP-1, inhibition of AR could halt such events. Indeed, a wide array of recent experimental evidence indicates that the inhibition of AR prevents oxidative stress-induced activation of NF-κB and AP-1 signals that lead to cell death or growth. Further, ARIs have been shown to prevent inflammatory complications such as sepsis, asthma, cancers (colon, breast, prostate, and lung), metastasis, and uveitis in animal models. The new in vitro and in vivo data have provided a basis for investigating the clinical efficacy of new ARIs with improved profiles as potential therapeutic agents in preventing inflammatory complications other than diabetes. The discovery of such new insights for this old enzyme could have considerable importance in envisioning potential new therapeutic strategies for the prevention and treatment of inflammatory diseases, thereby igniting a renewed interest in the field of ARIs (Table 2) [269].

## Figures and Tables

**Table 2:** A select list of aldose reductase (AR) inhibitors. Different categories (types, and subtypes) of AR inhibitors have been developed and tested both in experimental animals as well as in human subjects.

Categories (Types/Subtypes)	AR Inhibitors	Reference(s)
Naturally Occurring	D-saccharic acid 1,4—lactone Resveratrol Vitamin K1	[21,22,23]
Flavanone Glucoside	Hesperidin B-Glucogallin	[24,25,26,27,28]
Alkaloids	Berberine Palmatine Coptisine Iateorrhizine	[29,30,31,32,33,34,35,36,37,38,39,40,41,42,43]
Spirohydantoin	Sorbinil Fidarestat (SNK-860)	[44,45,46,47,48,49,50,51,52]
Acetic Acid	Alrestatin	[53,54,55,56,57,58,59,60,61]
Carboxyl Acid	Tolrestat Epalrestat Ponalrestat	[62,63,64,65,66,67,68,69,70,71,72,73,74,75,76,77,78,79,80,81,82,83,84,85,86,87,88,89,90]
Flavanoids	Quercetin	[29,37,91,92,93,94,95,96,97,98]
Spiroscinimide	Ranirestat (AS-3201) Zopolrestat NZ-314 Ponalrestat (z)2-(5-(4-methoxybenzylidine)-2,4-dioxothiazolidin-3-yl) acetic acid Zenarestat	[50,61,81,82,83,87,88,90,99,100,101,102,103,104,105,106,107,108,109,110,111,112,113,114,115,116,117,118,119,120,121,122,123,124,125,126,127,128,129]
